# Improvement of MBBR-MBR Performance by the Addition of Commercial and 3D-Printed Biocarriers

**DOI:** 10.3390/membranes13080690

**Published:** 2023-07-25

**Authors:** Dimitra C. Banti, Petros Samaras, Eleni Kostopoulou, Vassiliki Tsioni, Themistoklis Sfetsas

**Affiliations:** 1 QLAB Private Company, Research & Development, Quality Control and Testing Services, 57008 Thessaloniki, Greece; e.kostopoulou@q-lab.gr (E.K.); vtsioni@q-lab.gr (V.T.); tsfetsas@q-lab.gr (T.S.); 2 Department of Food Science and Technology, School of Geotechnical Sciences, International Hellenic University, 57400 Thessaloniki, Greece; samaras@ihu.gr

**Keywords:** MBBR-MBR, biofilm, 3D-printed biocarriers, Kaldnes K1 biocarriers, 13X-halloysite biocarriers, membrane fouling, SMP, EPS, colloidal particles, wastewater treatment

## Abstract

Moving bed biofilm reactor combined with membrane bioreactor (MBBR-MBR) constitute a highly effective wastewater treatment technology. The aim of this research work was to study the effect of commercial K1 biocarriers (MBBR-MBR K1 unit) and 3D-printed biocarriers fabricated from 13X and Halloysite (MBBR-MBR 13X-H unit), on the efficiency and the fouling rate of an MBBR-MBR unit during wastewater treatment. Various physicochemical parameters and trans-membrane pressure were measured. It was observed that in the MBBR-MBR K1 unit, membrane filtration improved reaching total membrane fouling at 43d, while in the MBBR-MBR 13X-H and in the control MBBR-MBR total fouling took place at about 32d. This is attributed to the large production of soluble microbial products (SMP) in the MBBR-MBR 13X-H, which resulted from a large amount of biofilm created in the 13X-H biocarriers. An optimal biodegradation of the organic load was concluded, and nitrification and denitrification processes were improved at the MBBR-MBR K1 and MBBR-MBR 13X-H units. The dry mass produced on the 13X-H biocarriers ranged at 4980–5711 mg, three orders of magnitude larger than that produced on the K1, which ranged at 2.9–4.6 mg. Finally, it was observed that mostly extracellular polymeric substances were produced in the biofilm of K1 biocarriers while in 13X-H mostly SMP.

## 1. Introduction 

Using Moving Bed Biofilm Reactors (MBBR) is the most simple and commonly used method for applying biocarriers during aerobic wastewater treatment [1]. However, certain limitations of MBBRs have led to the use of a hybrid model in which MBBRs are combined with other advanced wastewater treatment technologies, such as membrane bioreactors (MBR). The combination of MBBR-MBR was found to be more effective than MBR in terms of pollutant removal [2]. The advent of MBBR and MBR in the latter part of the 20th century has revolutionized research in the field of wastewater treatment [2,3,4]. The use of MBBR-MBR processes has shown promising results for the circular economy due to their high nutrient removal and recovery potential [3]. ΜΒΒR-MBR processes are financially and environmentally competitive methods, as they do not require the constant addition of costly reagents and they do not produce dangerous residuals [1]. This is, therefore, an advanced low-cost technology; while at the same time, it is also simple, reliable as well as stable to use, and it allows all processes to take place in one tank [5]. Despite the multiple advantages of MBBR-MBR use, there is one important disadvantage. In MBR technology, membrane fouling occurs, which is the unwanted deposit of suspended particles, colloidal and soluble components of mixed liquor on the surface and on the inside of the filtration membrane pores [6,7]. This phenomenon causes the transmembrane pressure to increase something that reduces the MBR performance resulting in the increase of operating and maintenance costs. 

Some of the main components of the activated sludge, which are also the most important membrane foulants, are the Soluble Microbial Products (SMP) and the Extracellular Polymeric Substances (EPS) [8]. The production of SMP and EPS is a typical process for microorganisms in their natural environment. SMP and EPS comprise a gel-like biofilm matrix that is particularly hydrated and often electrically charged, in which microorganisms are integrated and immobilized [9]. The mixed liquor of activated sludge also contains colloidal components, which, according to recent studies, are also an important membrane foulant [10]. 

At present, freely moving submerged biocarriers in aeration tanks are used in modern MBBR methods, combining two different processes: the processes of attached and suspended biomass growth. With this technology, the processes of biodegradation of organic pollutants, nitrification, denitrification, and ammonia removal are carried out [11,12]. In MBBR, each biocarrier increases the wastewater treatment performance by providing a protected surface for the growth of autotrophic and heterotrophic microorganisms and thus achieving high rates of degradation. For the MBBR to function properly, a steady-state biofilm should be formed on the surface of the biocarriers [13]. 

Various methods have been studied and used to deal with the issue of filtration membrane fouling, one of which is the addition of biocarriers. Biocarriers are added with the aim of performing an immediate abrasion of the membrane surface as they move in the membrane tank due to aeration, as long as they are put in the membrane tank, and on the other hand, they are added with the aim of reducing the sludge metabolism products, meaning the SMP and EPS, due to their attachment to the biofilm. Lee et al. (2021) [14] used granule-activated carbon biocarriers combined with Kaldnes K3 biocarriers modified by inserting a sponge cube and found that the additional internal recirculation improved the effluent quality further, by increasing the nitrogen removal. Membrane filterability improved as well, by reducing the cake layer resistance. Sun et al. (2021) [15] used K1 commercial biocarriers and produced an effluent of excellent quality. Moreover, the fouling of the ceramic membrane was reduced, a result attributed among others to the fluidized biocarriers, reducing energy consumption by 44%.

Biofilm thickness is one of the main parameters used when evaluating the MBBR performance. Studies have suggested that in a more than 700 μm thick biofilm nitrogen removal cannot be sustained due to the lack of substrate in the deep anaerobic layer. A critical parameter that determines the efficiency of MBBR technology is the appropriate design of the biocarrier [16]. The optimal biocarriers should have a large specific surface per volume unit. Other biocarrier characteristics that significantly affect the MBBR performance are the material they are made of, their surface characteristics, their orientation, the distance among the pores, and their geometry [17,18]. The large specific surface and the strong interactions of the bacterial surface are the two main criteria for the creation of high-performing biocarriers. Large specific surface area may increase volumetric loads of biomass, while strong bacterial surface interactions may prevent biofilm detachment caused by external hydraulic forces [19].

3D printing technology is the solution to the problem of designing the optimal biocarrier by offering flexibility in its design and in the selection of its manufacturing material. A first attempt to construct 3D-printed biocarriers and use them in an MBBR unit was made by Elliott et al. (2017) [18] and by Tang et al. (2017) [20] in which the efficiency of this technology for the improvement of biocarrier characteristics in MBBRs was proven. Dong et al. (2015) [12], fabricated a series of 3D biocarriers in the shape of hollow honeycomb spherical structures for COD and NH_3_ removal. Elliot et al. (2017) [18] succeeded in increasing the NH_3_ removal rate by 1620 ppm/d as compared to common biocarriers by creating spherical biocarriers with a larger specific surface area. There are also promising results in current studies regarding the unique advantages of 3D printing for the fabrication of biocarriers with the purpose of the production of biofilm highly loaded with dry mass and/or bio-activities. Chioti et al. (2022) [21] have found a remarkable wastewater treatment efficiency when studying the performance of Kaldnes K1 biocarriers and various 3D-printed 13X biocarriers in aerobic wastewater treatment lab reactors of an active volume of 150 mL.

The aim of this research work was to study the effect of commercial Kaldnes K1 biocarriers and 3D-printed biocarriers, fabricated from 13X zeolite and halloysite, on the efficiency and the fouling rate of a semi-pilot scale MBBR-MBR unit during municipal wastewater treatment. 

## 2. Materials and Methods

### 2.1. MBBR-MBR Set-Up and Operating Conditions

**Figure 1:**
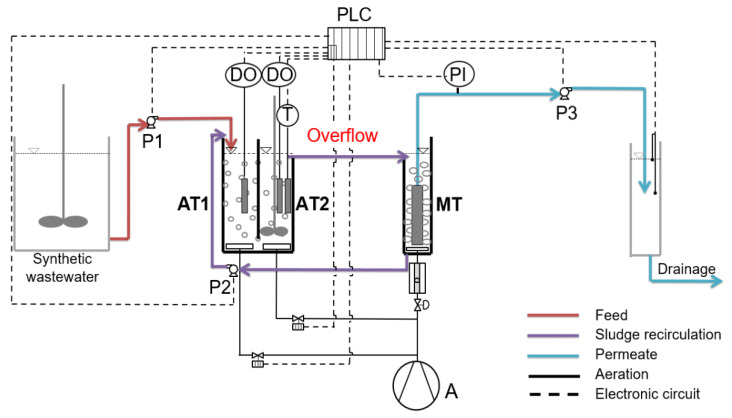
ΜΒΒR-MBR flow diagram in which: AT1: 1st aerated tank (V_AT1_ = 5 L), AT2: 2nd aerated tank (V_AT2_ = 5 L), MT: membrane tank (V_MT_ = 5 L), A: air Compressor, DO: dissolved oxygen measuring device and PLC: programmable logic controller, T: temperature measurement, PI: pressure indicator, P1, P2, P3: peristaltic pumps.

**Figure 2:**
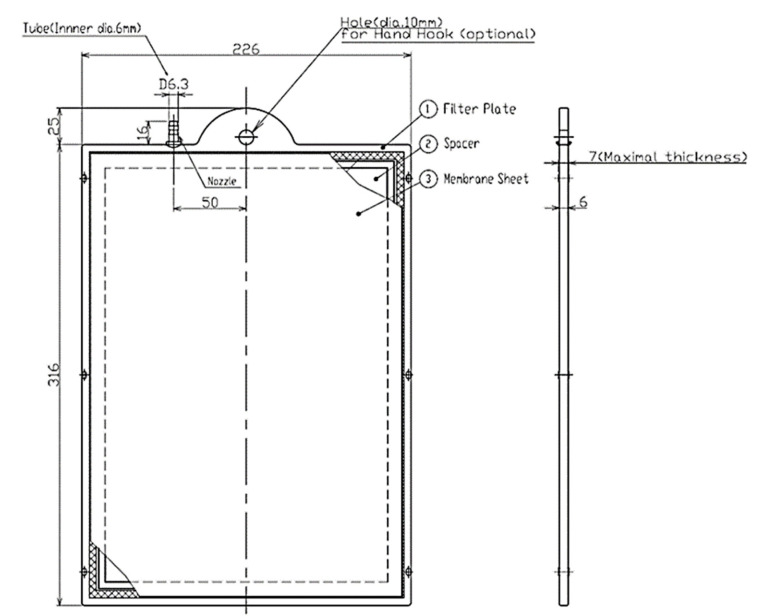
Geometry of the A4 flat sheet microfiltration membrane module designed by the manufacturer (Kubota).

For the purposes of this research, a semi-pilot MBBR was used combined with an MBR, with a total active volume of 15 L, the flow diagram of which is shown in Figure 1. The unit consisted of a synthetic wastewater storing tank, two aerated tanks of the same volume one after the other, and a membrane tank. In the membrane tank, a hydrophilic flat sheet A4 microfiltration membrane, type H-203, was submerged (Kubota, Osaka, Japan). The membrane sheet was held on each side of the membrane panel. Treated wastewater permeated through the membrane sheet and spacers to come out via a nozzle on the top. The membrane sheet, the geometry of which is presented in Figure 2, was made from chlorinated polyethylene (filtration agent) with a nominal (maximum) pore size of 0.4 μm. The effective membrane area was 0.11 m^2^, the dry weight was 0.4 kg and the clean water initial permeate flow was 120 mL/min for a water temperature of 20 °C and filtration pressure of 5 kPa. 

At the bottom of the membrane tank, intense aeration was applied, so that the cake layer that was formed on the membrane surface is removed. The filtration flow was intermittent with 10 min of operation and 2 min of pause. The synthetic sewage was prepared twice a week and had the following composition: glucose (500 mg/L), corn starch (500 mg/L), NH_4_Cl (200 mg/L), peptone (56 mg/L), KH_2_PO_4_ (53 mg/L), MgSO_4_·7H_2_O (18 mg/L), MnSO_4_·H_2_O (7.4 mg/L), FeSO_4_·7H_2_O (1.1 mg/L), and NaHCO_3_ (240 mg/L) [7,10]. At the start of the experiment, activated sludge from a wastewater treatment plant in Thessaloniki was added to the MBBR-ΜΒR tank for the acclimatization of the synthetic sewage. 

The MBBRs consisted of 2 dissolved oxygen measuring devices, 3 peristaltic pumps, an air compressor, a thermometer, and a PLC system (Eutech Instruments), which was used for documenting and setting the operation parameters with the help of SCADA software version 7.0 (Simantec, Siemens, Munich, Germany). The parameters documented and set via the SCADA software were: influent (Q_IN_ = 0.9 L/h), recirculation (Q_r_ = 2.1 L/h), effluent (Q_OUT_ = 1.1 L/h), temperature, dissolved oxygen concentration in the two aerated tanks, and transmembrane pressure drop. The temperature in the MBBR-MBR units was maintained relatively constant at 20–22 °C since the units were located in an indoor air-conditioned laboratory facility, while the temperature was recorded every minute in the SCADA software.

**Figure 3:**
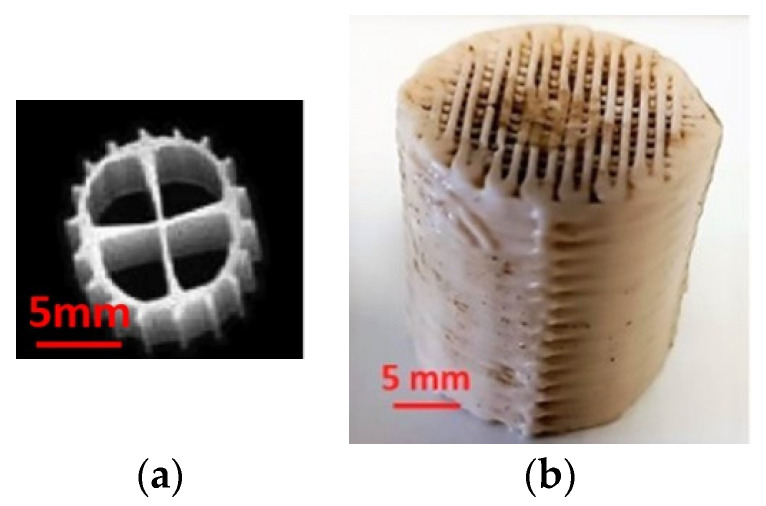
(**a**) Commercial Kaldnes Κ1 biocarriers and (**b**) 3D-printed biocarriers fabricated with 13Χ and halloysite.

Continuous flow operation of the reactors took place over a period exceeding one month, during which extended operation of the units was monitored, allowing the daily repetition of the experimental conditions of the unit and the verification of the reproducibility of the parameters and the processes. The MBBR-MBR was operated for the following three cases: No biocarriers were added to the unit in the first experiment and therefore it will be hereinafter referred to as control MBBR-MBR. During the second experiment, commercial Kaldnes K1 biocarriers with an active volume of 1 L were added to the first aerated tank of the MBBR-MBR unit (Figure 3a). This experiment will be hereinafter referred to as MBBR-MBR K1. Finally, 3D-printed biocarriers manufactured with 13X zeolite, one type of aluminosilicate crystal, and halloysite (Figure 3b), having an active volume of 1 L, were added in the first aerated tank of the MBBR-MBR unit during the third experiment. This experiment will be hereinafter referred to as MBBR-MBR 13X-H. The percentage of biocarriers added was equal to 20% of the first aerated tank (AT1) of 5 L active volume (Figure 1).

In all three MBBR-MBR units, synthetic wastewater with a similar COD inflow amount (779 ± 85 mg/L for control MBBR-MBR, 772 ± 98 mg/L for MBBR-MBR Κ1, and 780 ± 84 mg/L for MBBR-MBR 13X-H) was used. It was also made sure that the activated sludge mixed liquor contained a similar amount of Mixed Liquor Suspended Solids (MLSS) in all three units (5.9 ± 0.4 g/L for control MBBR-MBR, 6.2 ± 0.7 g/L for MBBR-MBR Κ1, and 5.8 ± 0.7 g/L for MBBR-MBR 13X-H). To maintain the MLSS values, regular measurements took place and, depending on the results, activated sludge was either added or removed. The dissolved oxygen in the two aerated tanks (AT1 and AT2) was kept at 2.5 mg/L in all three MBBR-MBRs so that the aerobic biodegradation of the wastewater is efficient. Finally, in all three experiments, there were also similar F/M ratio values (0.19 g COD/g MLSS/d for control ΜΒΒR-MBR, 0.18 g COD/g MLSS/d for MBBR-ΜΒR Κ1, and 0.19 g COD/g MLSS/d for MBBR-ΜΒR 13X-H). The above were decided with the purpose of enabling the comparison of the three experiments. 

### 2.2. Biofilm Extraction Method

Sampling of biocarriers from the MBBR-MBR unit and biofilm extraction from their surfaces were regularly performed with the purpose of determining the dry mass, the MLSS, SMP, and EPS. The extraction of biofilm was carried out using the Mandakhalikar et al. (2018) [22] method, which includes placing the biocarrier sample in 10 mL of deionized water, stirring it in a vortex device for 1 min, placing it in an ultrasonic device for 2 min, and then repeating the stirring in a vortex device for another minute. This methodology contributed to the optimal removal of the biofilm. The same procedure was retrieved for all the biocarrier samples for both K1 and 13X-H biocarriers aiming to conclude with comparable results.

### 2.3. Printing Methodology of the 3D-Printed Biocarriers with 13X Zeolite and Halloysite

According to [23], the mixing of 13X zeolite with the inorganic halloysite nanotubes led to a greater specific surface area, total pore volume, as well as micro- and meso-pore volume, compared to the addition of other inorganic binders or 13X alone, as well as compared to other material combinations [24]. This can be attributed to the nanotubes having the ability to facilitate the dispersion of molecules within their structure, thus enhancing the degree of dispersion of 13X zeolite [23]. Therefore, this paste synthesis was preferred, as it could potentially promote biominfilm formation on the printed carriers. The preparation of the printing paste included the mixing of the ceramic material (13X), the inorganic (halloysite), and the organic binders in a mortar to the point where the mixture of solid content was homogenized. Afterward, deionized water was mixed with the colloidal silica. To ensure the optimal density of the printed ceramic paste and prevent excessive shrinking during the drying and calcination processes, it was necessary for the paste to have a content of solids exceeding 50%. Finally, the mixture of liquid content was gradually added to the mixture of solids, at the same time, it was fused with a pestle until a paste was formed to achieve homogenization. To remove all aggregates and grains from the paste, the mixture was sieved in a 45 μm hole-diameter sieve. Table 1 below shows the zeolite and inorganic binders’ proportions. 

**Figure 4:**
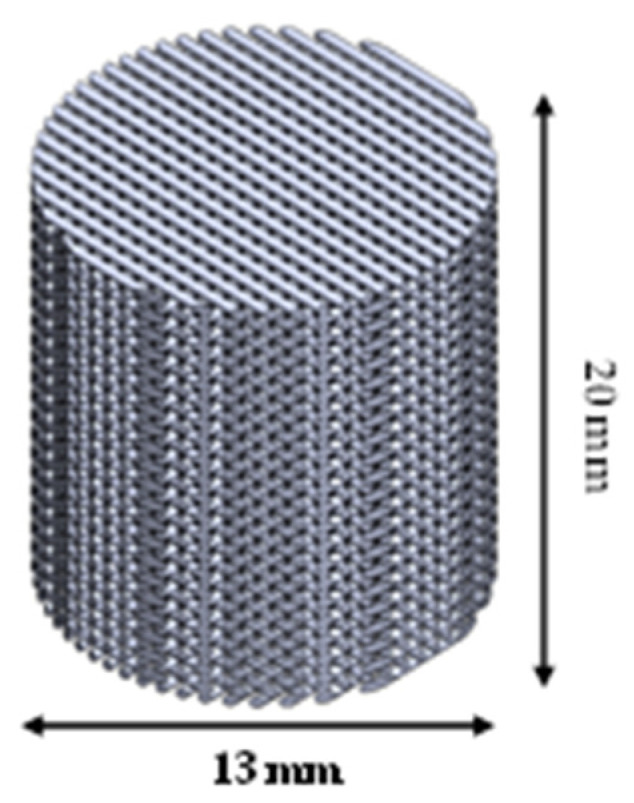
Geometry and dimensions of the 3D printed 13X-H biocarriers as determined in AutoCAD.

Centrifugation took place to ensure the removal of any air bubbles, transferred to the syringe, and finally to the printer. Then, the process of preparing the printer took place. This process included the creation of the biocarriers 3D geometry, during which parameters like the shape, the recurring geometric pattern, the height of each layer, the nozzle diameter, the pressure, and the printing speed were determined. A cylinder was chosen as the recurring geometric pattern for the biocarriers and the dimensions of the biocarrier were: 13 mm width, 13 mm length, 20 mm height, and 4.8564 g weight. This structure was particularly opted as it combines practicality for the 3D printing process, not requiring additional support material, and has a higher specific surface area compared to other designed geometries. Moreover, it constitutes a simple, representative model of a regular, straight-channel (beam-shaped, log-pile) configuration [25]. This configuration presents an open lattice and stable scaffold build-up by laying down the fibers in a 0°/90° pattern with the fiber diameter equal to the outlet diameter of the selected nozzle (ca. 0.8 mm) and inter-fiber spacings equal to the fiber diameter. The reasoning behind that selection is to enable the validation and optimization of the biocarrier geometry that provides mechanical stability, low pressure drop, high surface area, and high transfer characteristics for operating a fixed bed system. A representation of the 3D model geometry is shown in Figure 4. An in-house Computer Numerical Control (CNC) printer was used for the printing.

### 2.4. Mechanical Properties of the 3D-Printed 13X-H Biocarriers

The mechanical properties of the 3D-printed 13X-H biocarriers were extensively investigated by members of this research team comparing them with various zeolite–binder combinations fabricated through 3D printing with improved properties and increased performance [24]. It is worth highlighting that 13X-Halloysite nanotube biocarriers presented the highest hardness, equal to 46 ± 5 MPa, compared to 4 other zeolite–binder combinations. In detail, 13X-Bentonite carriers showed a hardness of 44 ± 5 MPa, ceramic material ZSM-5-Bentonite presented 38 ± 8 MPa, ZSM-5-Montmorillonite 38 ± 2 MPa, and ZSM-5-Halloysite 29 ± 6 MPa [24]. Τhis was an additional reason for choosing the 13X-H biocarriers as the optimum ones for this research work.

### 2.5. Determination of the Physicochemical Parameters

The physicochemical parameters used in the influent and effluent wastewater characterization (COD, Total N, NH_4_-N, and NO_3_-N) were determined using Hack–Lange LCK kits, along with a DR-2800 spectrophotometer. Mixed liquor suspended solids (MLSS) were measured according to standard methods [26]. The measurements were performed regularly to evaluate the progress of the experiments and the efficiency of ΜΒΒR-MBR treatment. 

Regular measurements, documentation, and setting of dissolved oxygen also took place, using a Greisinger OXY 3610 MP measuring electrode.

Samples were taken from the membrane tank and the effluent on a regular basis to measure the SMPs and EPS. For SMP and EPS extraction, a natural thermal extraction method was used [6,27]. Then, the extracted SMPs and EPS in the form of proteins were measured using the modified Lowry method and repeating it three times [28], while the SMPs and EPS in the form of carbohydrates were measured using the photometric method proposed by Dubois et al. (1956) [29] and repeating it two times. The protein calibration curve was prepared using Bovine Serum Albumin (ΒSA, Sigma Aldrich, St. Louis, MO, USA) and the carbohydrates calibration curve was prepared using glucose (Panreac, Barcelona, Spain).

Dynamic light scattering (Brookhaven Instruments, Holtsville, NY, USA) was used to determine the size distribution of the less than 1 μm colloidal particles of the membrane tank and the effluent. Static light scattering (Mastersizer, Malvern, UK) was used to determine the size distribution of aggregates with a diameter larger than 10 μm. Activated sludge and effluent were also observed under a Light Sheet Microscope (LSM, Observer Z1, Zeiss, Oberkochen, Germany) and filamentous index (FI) evaluation was also performed [30,31] in order to measure the population of filamentous microorganisms in the activated sludge mixed liquor. The microscope images were edited using ZEN software version 3.6 (ZEISS Group, Singapore).

### 2.6. DNA Extraction and 16S rRNA Gene Amplicon Sequencing

DNeasy PowerSoil Pro Kit (QIAGEN, Hilde, Germany) was employed to extract genomic DNA from the biofilm suspensions according to the manufacturer’s guidelines. Library preparation for sequencing of the 16S rRNA gene was carried out based on the standard 16S Metagenomic Sequencing Library Preparation protocol (IlluminaTM, Inc., San Diego, CA, USA). Specifically, the V3-V4 hypervariable regions of the 16S rRNA gene were targeted using the 341f/805r primer pair (341f 5′-CCTACGGGNGGCWGCAG-3′, 805r 5′-GACTACHVGGTATCTAATCC-3′). The resulting DNA libraries were quantified with a Qubit™ 4 Fluorometer (Thermo Fisher Scientific, Waltham, MA, USA), and their size was confirmed by electrophoresis on a 1.5% agarose gel. The libraries were equimolarly pooled, and their concentration was assessed by quantitative PCR using the QIAseq Library Quant Assay Kit (QIAGEN, Germany). The pooled library was spiked with 25% phiX control library (Illumina Inc., San Diego, CA, USA), denatured, and diluted to a final concentration of 6 pM. Sequencing was carried out on an Illumina MiSeqTM platform with either the MiSeq Reagent Nano Kit version 2 (500-Cycle) or the MiSeq Reagent Kit version 3 (600-Cycle) chemistry for a paired-end, 2 × 250-bp or 2 × 300 cycle run.

### 2.7. Bioinformatics

To analyze the data obtained from the sequencing, the Quantitative Insights Into Microbial Ecology 2 (QIIME2) software, version 2022.2 [32] was used. On average, a total of 50,000 raw reads per sample were sequenced. Initially, during the demultiplex and trimming steps, the low-quality readings were removed, such as reads up to Q30 and reads with unsatisfactory length and chimeras were also removed with the DADA2 algorithm [33], resulting in the construction of Amplicon Sequence Variants (ASV). Taxonomic annotation of the reads was performed using the SILVA reference database (SSU, release 138) [34]. For alpha diversity analysis, the q2-diversity plugin was utilized to calculate several metrics, such as Shannon, Simpson, and Fisher indices representing species diversity. The Chao1 and ACE indices representing species richness were calculated. To evaluate the similarity of microbiota from different groups, principal coordinates analysis (PCoA) plots using Emperor for each beta diversity were generated, whilst Faith’s Phylogenetic Diversity and weighted and unweighted UniFrac distances were reported. 

## 3. Results

### 3.1. Evaluation of the MBBR-MBR Performance for the 3 Units

**Figure 5:**
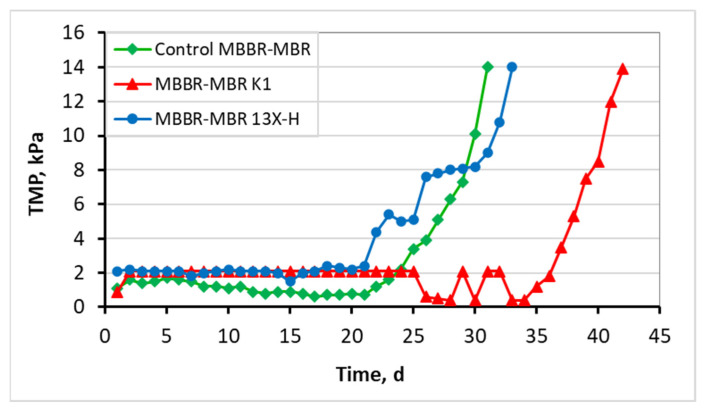
Transmembrane pressure (TMP) in relation to operating time for the control MBBR-MBR, the MBBR-MBR K1, and the MBBR-MBR 13X-H.

The MBBR-MBR units were operating under a Food/Microorganisms (F/M) loading of 0.18–0.19 g COD/g MLSS/d, which is within the desirable value range for efficient wastewater treatment. The measured transmembrane pressure (TMP) versus operation time is presented in Figure 5 for all three MBBR-MBR units. As shown in Figure 5, total membrane fouling was reached on the 31st day of unit operation in the control MBBR-MBR. With the addition of commercial Kaldnes K1 biocarriers, a remarkable improvement in filtration was observed, and membrane fouling took place on the 42nd day of unit operation. With the addition of 13X-H 3D-printed biocarriers, the total fouling took place on the 33rd day of unit operation. Therefore, the addition of 13X-H biocarriers allowed a membrane filtration efficiency of the same degree as in the control MBBR-MBR, not showing any substantial improvement or deterioration. 

**Figure 6:**
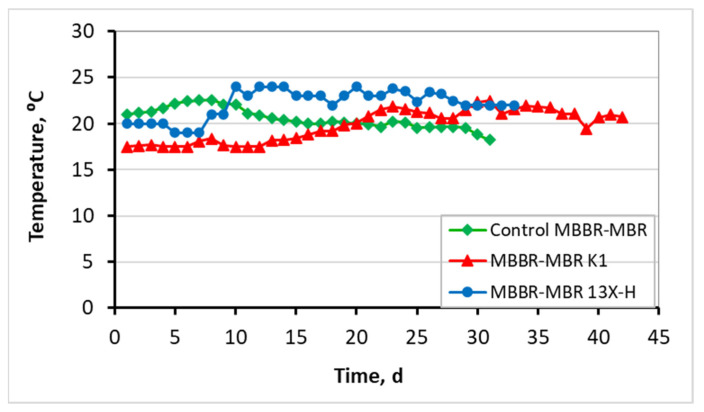
Temperature in relation to operating time for the control MBBR-MBR, the MBBR-MBR K1, and the MBBR-MBR 13X-H.

**Figure 7:**
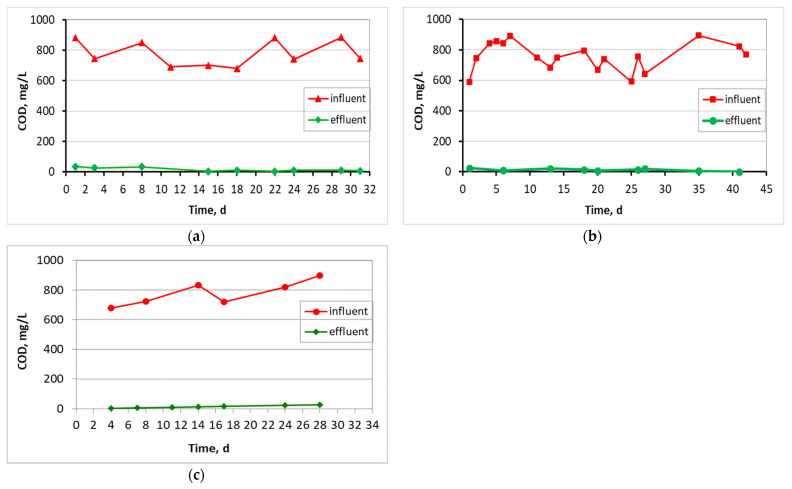
COD in relation to operating time for (**a**) the control MBBR-MBR, (**b**) the MBBR-MBR K1, and (**c**) the MBBR-MBR 13X-H.

Figure 6 shows the temperature that was kept fairly constant for the three units, with an average temperature of about 20–22 °C, as the units were placed indoors in an air-conditioned lab room. Then, Figure 7 shows COD results for the influent and effluent wastewater. Influent COD in the control MBBR-MBR was equal to 779 ± 85 mg/L, in the MBBR-MBR K1 equal to 772 ± 98 mg/L, and in the MBBR-MBR 13X-H 780 ± 84 mg/L. In the effluent, COD was decreased efficiently at 16 ± 13 mg/L, 13 ± 8 mg/L, and 20 ± 7 mg/L at the three MBBR-MBR units, respectively. 

**Figure 8:**
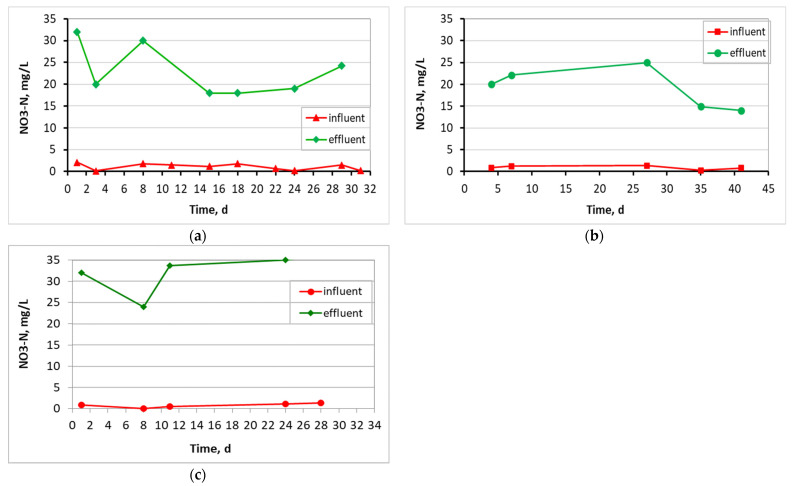
NO_3_-N in relation to operating time for (**a**) the control MBBR-MBR, (**b**) the MBBR-MBR K1, and (**c**) the MBBR-MBR 13X-H.

Figure 8 presents NO_3_-N results for the influent and effluent wastewater in all three MBBR-MBR units. Specifically, NO_3_-N in the influent wastewater of the control MBBR-MBR unit was equal to 1.1 ± 0.7 mg/L, for the MBBR-MBR K1 unit was 0.9 ± 0.4 mg/L, and equal to 0.8 ± 0.5 mg/L for the MBBR-MBR 13X-H. NO_3_-N value effectively increased as a result of the nitrification carried out during the treatment of wastewater in the units to 23 ± 5.4 mg/L at the control MBBR-MBR, 19 ± 4.7 mg/L at the MBBR-MBR K1, and 32 ± 4.8 mg/L at the MBBR-MBR 13X-H. 

**Figure 9:**
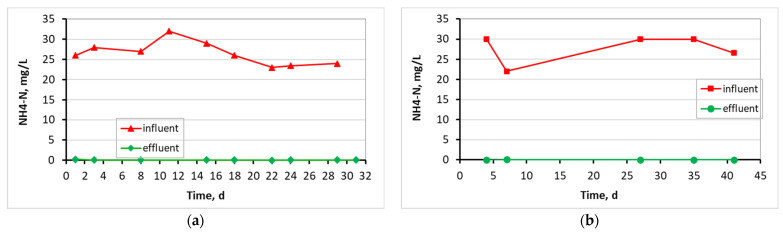

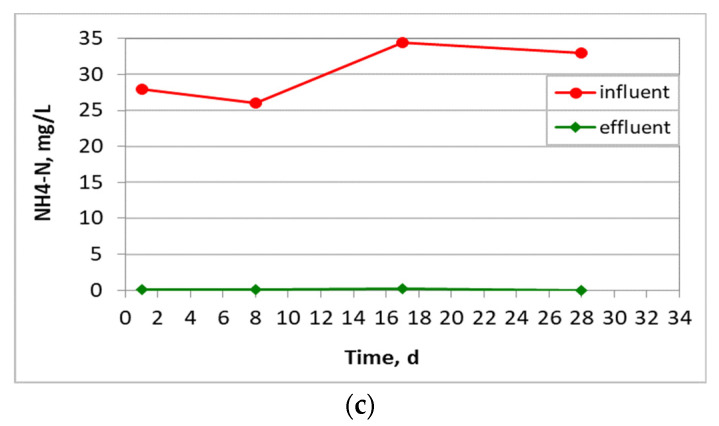
NH_4_-N in relation to operating time for (**a**) the control MBBR-MBR, (**b**) the MBBR-MBR K1, and (**c**) the MBBR-MBR 13X-H.

According to Figure 9, where NH_4_-N removal is presented for the three MBBR-MBR units, the influent values of 26 ± 2.9 mg/L (control MBBR-MBR), 28 ± 3.5 mg/L (MBBR-MBR K1), and 30 ± 4.0 mg/L (MBBR-MBR 13X-H) were zeroed to 0.05 ± 0.05 mg/L at the control MBBR-MBR, 0.03 ± 0.02 mg/L at the MBBR-MBR K1, and 0.09 ± 0.11 mg/L at the MBBR-MBR 13X-H. 

**Figure 10:**
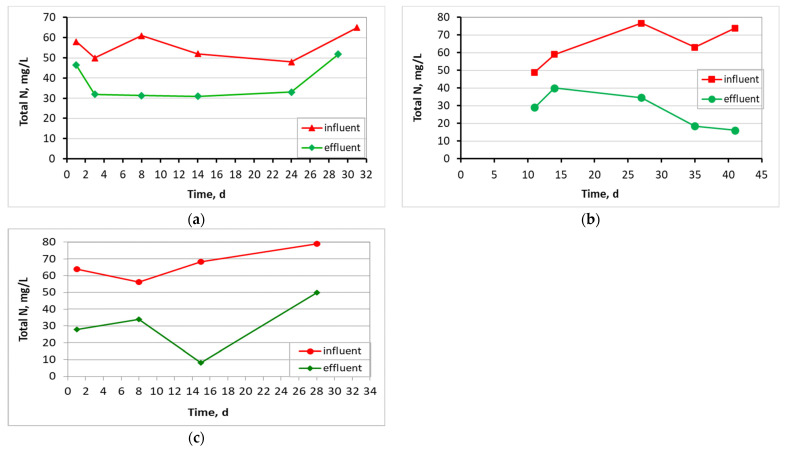
Total N in relation to operating time for (**a**) the control MBBR-MBR, (**b**) the MBBR-MBR K1, and (**c**) the MBBR-MBR 13X-H.

Finally, Figure 10 shows the total N removal of the units where, at the control MBBR-MBR, it decreased from an average value of 56 ± 6.7 mg/L in the influent wastewater to 40 ± 10 mg/L in the effluent; at the MBBR-MBR K1, from 64 ± 11 mg/L to 28 ± 10 mg/L; and at the MBBR-MBR 13X-H, from 67 ± 9.5 mg/L to 30 ± 17 mg/L. Therefore, total N removal was improved at the MBBR-MBR 13X-H compared to the control MBBR-MBR and was approximately the same as the MBBR-MBR Κ1. 

**Figure 11:**
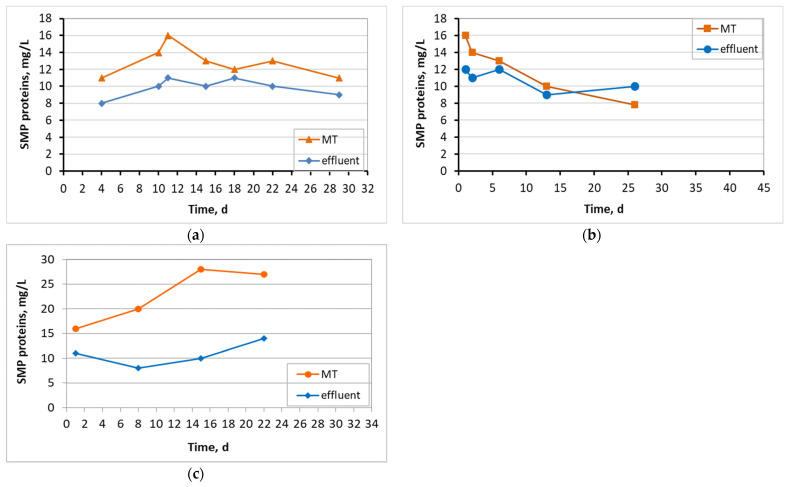
Concentration of SMP proteins in the membrane tank (MT) and effluent in relation to operating time for the (**a**) control MBBR-MBR, (**b**) MBBR-MBR K1, and (**c**) MBBR-MBR 13X-H.

**Figure 12:**
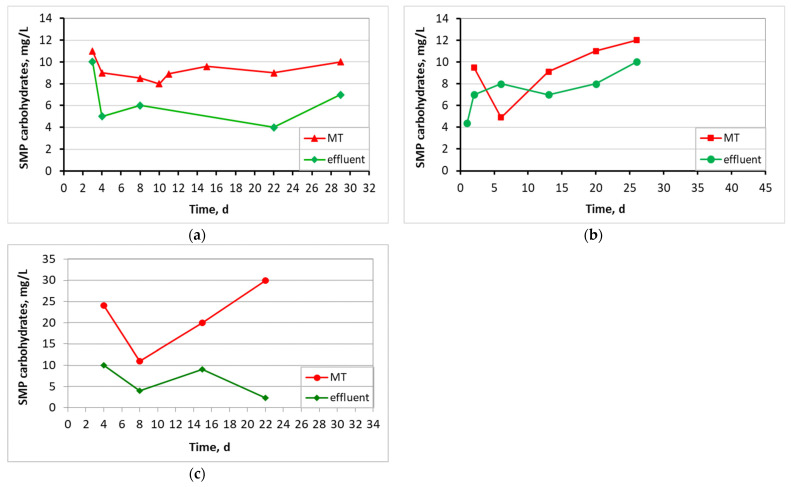
Concentration of SMP carbohydrates in the membrane tank (MT) and effluent in relation to operating time for the (**a**) control MBBR-MBR, (**b**) MBBR-MBR K1, and (**c**) MBBR-MBR 13X-H.

Figure 11 and Figure 12 present the SMP proteins and SMP carbohydrates concentration diagrams for mixed liquor in the membrane tank and the effluent in all three MBBR-MBR units.

**Figure 13:**
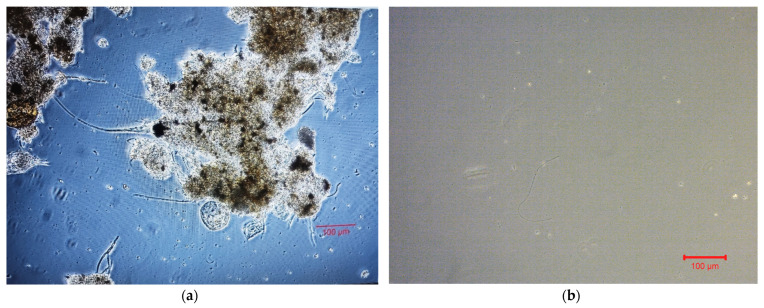
Standard images of optical microscope for (**a**) the mixed liquor and (**b**) the effluent.

**Figure 14:**
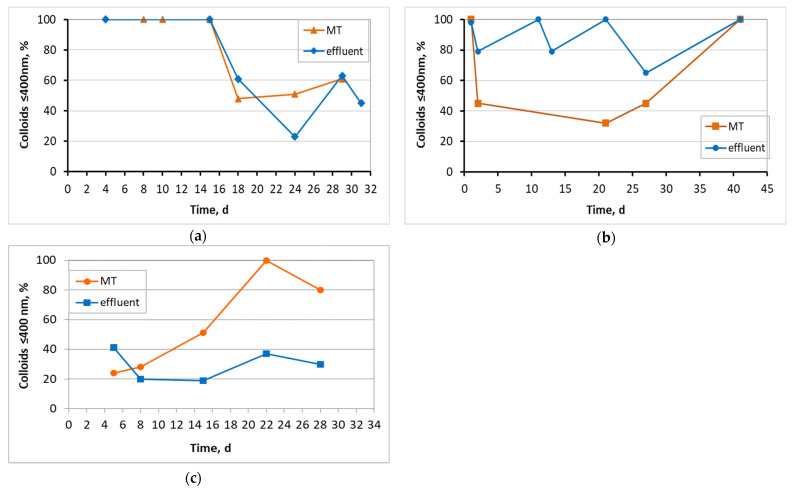
Percentage of colloidal particles with size ≤ 400 nm in relation to the operating time for the membrane tank (MT) and effluent in (**a**) the control MBBR-MBR, (**b**) the MBBR-MBR K1, and (**c**) the MBBR-MBR 13X-H.

Filamentous microorganisms protruding from the sludge flocs are observed in the mixed liquor photograph drawn by an optical microscope (Figure 13a). Figure 13b shows a typical optical microscope image of the effluent in MBBR-MBR units. The average aggregates size in the mixed liquor of the first aerated tank, according to measurements in Mastersizer, ranged up to 325 μm in control MBBR-MBR, to 139 μm in ΜBBR-MBR K1, and to 306 μm in MBBR-MBR 13X-H. Figure 14 shows the ≤400 nm colloidal particle rates for the mixed liquor in the membrane tank and for the effluent in all three MBBR-MBR units.

### 3.2. Biofilm Evaluation for the Kaldnes K1 Biocarriers

**Figure 15:**
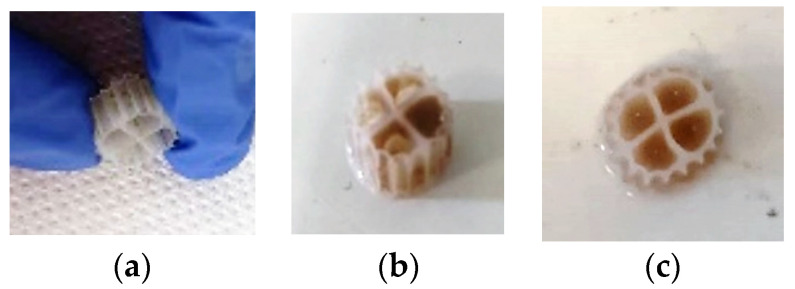
Biocarriers with the formed biofilm on the (**a**) 6th day, (**b**) 27th day, and (**c**) on the 48th day of the MBBR-MBR K1 operation.

Figure 15 shows the biocarriers and the biofilm that was developed on the inside of the biocarriers’ surfaces in relation to the operating time of the MBBR-MBR K1 unit. Table 2 shows the dry mass and MLSS concentration measurements in the biofilm that was developed on the surfaces of the biocarriers. The measurements were the result of the average biofilm value that was developed in three biocarriers per day of measurement.

**Figure 16:**
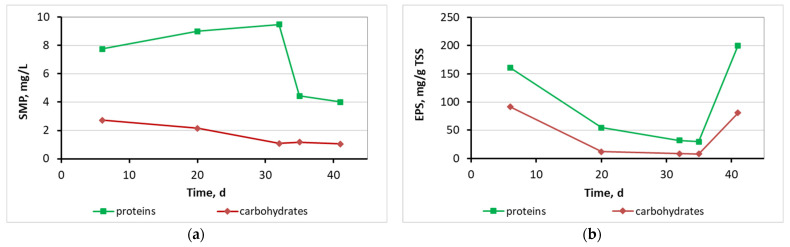
(**a**) SMP and (**b**) EPS protein and carbohydrates concentration in the biofilm of K1 biocarriers in relation to time.

Figure 16 presents the SMP and EPS protein and carbohydrate concentration for the biofilm of biocarriers in relation to time after biofilm extraction from four biocarriers per day. As illustrated in Figure 16, the concentration of SMP proteins was gently increasing from 8 to 9.5 mg/L until the 32nd day of the MBBR-MBR K1 operation, respectively, with the increase in biofilm. Their decrease on the 35th to 40th day to 4.5 and 4 mg/L, respectively, is linked to the biofilm detachment due to aeration. The EPS proteins and carbohydrates concentration fluctuated in a high average value of 90 mg/g TSS and 40 mg/g TSS correspondingly, while no correlation to the growing biofilm was observed.

### 3.3. Biofilm Evaluation for the 3D-Printed 13X and Halloysite Biocarriers

**Figure 17:**
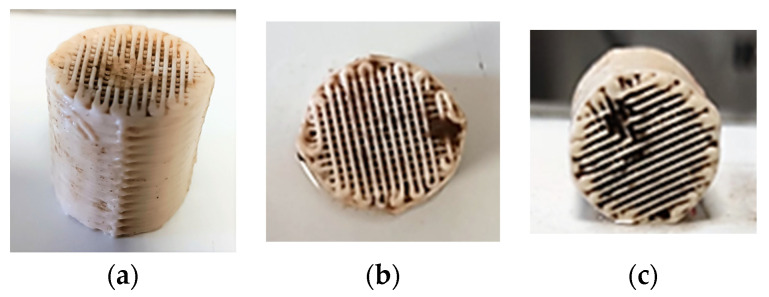
Biocarriers with the formed biofilm on the (**a**) 11th day, (**b**) 15th day, and (**c**) 24th day of the MBBR-MBR 13X-H operation.

Figure 17 shows the biocarriers and the biofilm that was developed on the inside of the biocarriers’ surfaces in relation to time. Table 3 shows the dry mass and MLSS concentration measurements in the biofilm that was developed on the surfaces of the biocarriers. The measurements were the result of the average biofilm value that was developed in three biocarriers per day of measurement.

**Figure 18:**
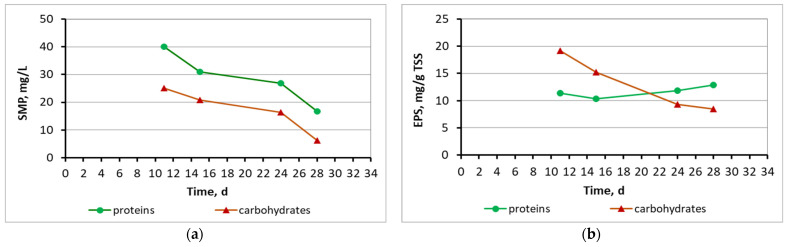
(**a**) SMP and (**b**) EPS protein and carbohydrates concentration in the biofilm of the 13X-H biocarriers in relation to time.

**Figure 19:**
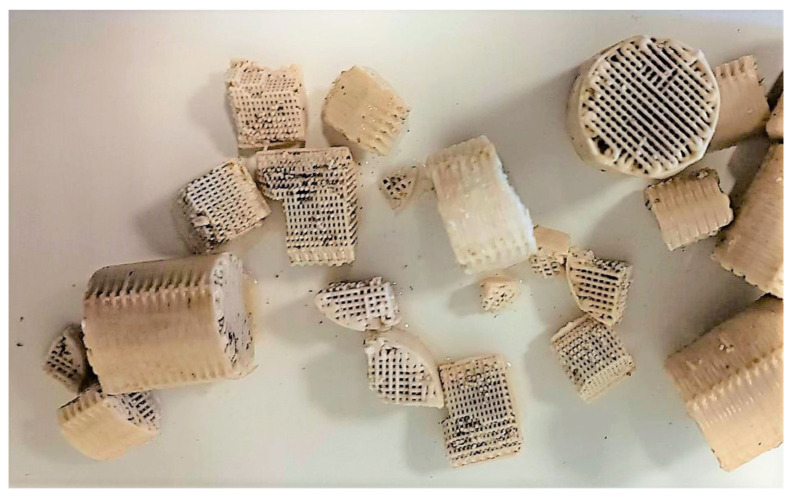
13X-H biocarriers’ fragments at the end of the experiment.

Figure 18 shows the SMP and EPS protein and carbohydrates diagrams for the biofilm of the 13X-H biocarriers in relation to operation time, after biofilm extraction from four biocarriers per day of measurement. SMP proteins reached an average value of 29 mg/L and SMP carbohydrates ranged at 17 mg/L on the 13X-H biofilm. EPS proteins reached a low average value of 12 mg/g TSS on the 13X-H biocarriers and EPS carbohydrates at 13 mg/g TSS. Finally, Figure 19 presents the fragments of 13X-H biocarriers at the end of the experiment.

### 3.4. Microbiome Analysis on Biofilm of Biocarriers via 16S rRNA Sequencing

**Figure 20:**
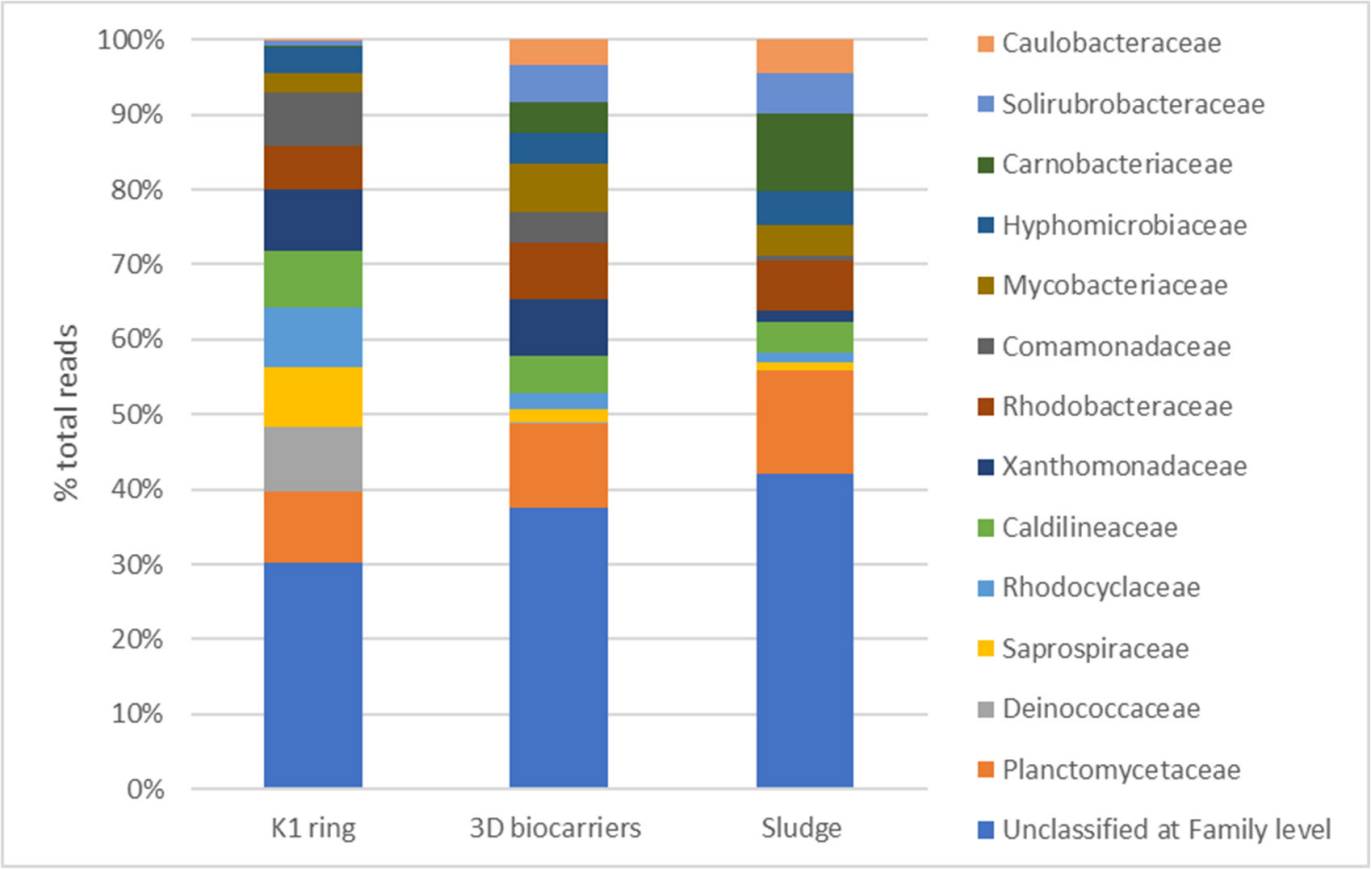
Relative abundance of the core families found on the biofilm of K1 ring and 3D-printed biocarriers at 30 days of operation.

**Figure 21:**
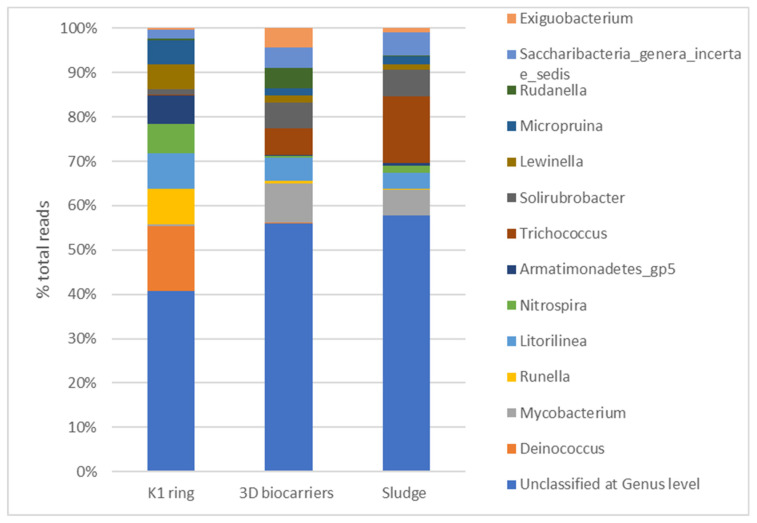
Relative abundance of the core genera found on the biofilm of K1 ring and 3D-printed biocarriers at 30 days of operation.

The findings from the microbiome analysis of the biofilm developed on K1 and 3D-printed biocarriers using 16S rRNA sequencing on day 30 of operation indicated that *Proteobacteria* (34.2–37.2%), *Actinobacteria* (9.6–19.3%), *Bacteroidetes* (8.4–16.9%), *Firmicutes* (2.8–9.9%), *Chloroflexi* (3.2–6.7%), and *Planctomycetes* (6–6.5%) were among the most abundant phyla. *Alphaproteobacteria* (19.4%), together with *Actinobacteria* (19.3%) dominated in the 3D-printed biocarriers, followed by *Gammaproteobacteria* (9.7%), *Planctomycetia* (6.5%), *Bacilli* (5.3%), and *Betaproteobacteria* (4.5%). *Alphaproteobacteria* (11.2%) were also dominant in the K1 ring, along with *Betaproteobacteria* (10.9%), *Actinobacteria* (9.6%), *Sphingobacteriia* (9%), *Gammaproteobacteria* (8.2%), and *Planctomycetia* (5.8%). As regards the families, the biofilm on 3D-printed biocarriers was mainly composed of *Planctomycetaceae* (6.5%), *Xanthomonadaceae* (4.4%), *Rhodobacteraceae* (4.3%), and *Mycobacteriaceae* (3.7%). Similarly, *Planctomycetaceae* (5.8%) and *Xanthomonadaceae* (5.1%) were also enriched in the K1 carrier; however, the overall profile was different, with *Deinococcaceae* (5.3%), *Saprospiraceae* (4.9%), *Rhodocyclaceae* (4.8%), *Caldilineaceae* (4.5%), and *Comamonadaceae* (4.2%) being the most abundant. At the genus level, 3D-printed biocarriers were mainly characterized by *Mycobacterium* (3.5%), followed by *Trichococcus* (2.3%), *Solirubrobacter* (2.2%), *Litorilinea* (2%), *Rudanella* (1.8%), and *Saccharibacteria* genera incertae sedis (1.8%). Conversely, in the biofilm of the K1 ring the major genera were *Deinococcus* (5.3%), *Runella* (3%), *Litorilinea* (2.9%), *Nitrospira* (2.4%), *Armatimonadetes* gp5 (2.3%), *Lewinella* (2%), and *Micropruina* (1.9%). The sludge obtained from the control MBBR-MBR unit exhibited a microbial composition more similar to the biofilm microbiota of the 13X-H biocarriers compared to that of the K1 ring biofilm. However, *Planctomycetaceae* (8.5%) and *Carnobacteriaceae* (6.4%), were found more enriched, while *Xanthomonadaceae* (1%) and *Comamonadaceae* (0.5%) were less abundant. The latter, interestingly, was also highly present in the K1 ring. Differentially abundant genera included *Trichococcus* (6.3%) and *Phenylobacterium* (2.7%), which were substantially more enriched in the sludge compared to the biocarriers, and *Nitrospira* (0.7%), which had higher abundance in the sludge than in the 13X-H biocarriers, though K1 ring showed the greatest development. Of note, the biofilm of 3D-printed biocarriers, 13X-H, included considerably higher numbers of *Rudanella* (1.8%) and *Exiguobacterium* (1.7%) in comparison with the other two conditions, while *Deinococcus* (5.3%) dominated solely in K1 ring. Figure 20 and Figure 21 show the most prevailing families and genera, respectively, detected within the biofilm of K1 and 3D-printed biocarriers, as well as in the control sludge.

## 4. Discussion

As shown in Figure 5, the transmembrane pressure (TMP) in the control MBBR-MBR was maintained lower than 2 kPa until the 24th day of unit operation, and total membrane fouling was reached on the 31st day of unit operation. With the addition of commercial Kaldnes K1 biocarriers, a remarkable improvement in filtration was observed, and membrane fouling took place 11 days later. More specifically, TMP was maintained lower than 2 kPa until the 36th day of unit operation and total membrane fouling took place on the 42nd day of unit operation. With the addition of 13X-H 3D-printed biocarriers, TMP increased by more than 2 kPa on the 21st day, while the total fouling took place on the 33rd day of unit operation. Therefore, the addition of 13X-H biocarriers allowed a membrane filtration efficiency of the same degree as in the control MBBR-MBR, not showing any substantial improvement or deterioration. Despite the fact that the addition of 13X-H biocarriers contributed to the successful growth and protection of a significant amount of biofilm on their surfaces, it was, nevertheless, not enough to improve membrane filtration. This is attributed to the fact that a small part of the biocarriers became fragmented due to the aeration in the tank, which led to the scattering of tiny particles of halloysite in the mixed liquor, particles that were most probably driven to the membrane pores and blocked them. Another reason for this result is the production of a large amount of SMP produced (Figure 18 and Figure 19), resulting from a great growth of biofilm on the biocarriers (Table 3). Other researchers have also observed such a result for other types of biocarriers in MBBR-MBR units [2,35]. 

As illustrated in Figure 6, the temperature was kept fairly constant for the three units, with the control ΜΒΒR-MBR at 21 ± 1.1 °C, ΜΒΒR-MBR K1 at 20 ± 1.7 °C, and MBBR-MBR 13X-H at 22 ± 1.6 °C. Based on Figure 7 and on the influent and effluent COD values, it can be concluded that there is an excellent effluent quality in all three ΜΒΒR-MBR units, with a COD removal rate of 98% for all units. NO_3_-N in the effluent of the unit was increased in control MBBR-MBR as much as it was increased in MBBR-MBR K1 (95%). The increase in NO_3_-N was slightly better in MBBR-MBR 13X-H as it was 97.5% (Figure 8). ΝH_4_-Ν concentration was significantly reduced reaching 0 mg/L, meaning that the removal rate reached approximately 100% in all three MBBR-MBRs (Figure 9). Finally, 29% of total N was removed from control MBBR-MBR, while 57% of total N was removed from MBBR-MBR Κ1, showing a clear improvement (Figure 10). Of total N, 55% was removed from MBBR-MBR 13X-H, meaning that the removal in MBBR-MBR 13X-H was better than in the control MBBR-MBR and slightly less than the MBBR-MBR Κ1. 

It can, therefore, be concluded that the processes of nitrification and denitrification were enhanced with the addition of K1 and 13X-H biocarriers in the MBBR-MBR unit in relation to control MBBR-MBR, as the MBBR incorporates the advantages of both suspended and attached growth process where microorganisms grow on biocarriers in the form of biofilm [36]. The biofilm formed on biocarriers includes the existence of anoxic/anaerobic inner layers and aerobic outer layers. Therefore, nutrient removal is accomplished in a single reactor reducing the land area requirement for wastewater treatment plants. An excellent effluent quality in relation to COD, NH_4_-Ν, and Total N removal, was mentioned in other studies as well [3,37]. Wastewater treatment performance similar to the one in the current experiment was observed with the only difference being the use of bigger filling ratios (0.35–0.67) as opposed to this experiment in which a 0.20 filling ratio was used.

Figure 11 and Figure 12 indicate the SMP protein and carbohydrate concentration in all three reactors. In control MBBR-MBR and in MBBR-MBR K1, it was found that a high concentration of SMP exits into the filtrate, something that was not observed in MBBR-MBR 13X-H, as a smaller amount of SMP exits into the filtrate. Most likely, this is due to the fact that, as discussed below, more biofilm is developed in 13X-H biocarriers than in K1 biocarriers. As a result, SMP is kept on the inside of the biofilm and prevents the SMP from exiting into the filtrate (Figure 18). This finding plays a significant role in the improvement of the filtrate membrane performance because the SMP is one of the most important membrane foulants. More specifically, in control MBBR-MBR the SMP protein concentration was 13 mg/L and the SMP carbohydrates concentration was 9.3 mg/L. In MBBR-MBR Κ1, the average values of SMP were similar to the values in control MBBR-MBR, with the protein concentration being 13 mg/L and the SMP carbohydrates concentration being 10 mg/L. In MBBR-MBR 13X-H, the average values of SMP increased by approximately 10 units as compared to the other experiments, with the SMP protein concentration being 23 mg/L and the SMP carbohydrate concentration being 21 mg/L. As mentioned before, this is due to the increased biofilm production in the 13X-H biocarriers. This phenomenon is, on the one hand, beneficial, as it improves the performance of wastewater treatment but on the other, it leads to an increase of the generated SPM, which are basic foulants for the filtration membrane. Similar results for other types of biocarriers in MBBR-MBR units were also found by other researchers [2,35], who tried to fix this issue by applying intermittent voltage in the membrane tank.

The filamentous index (FI) for the activated sludge, according to Figure 13a, ranged 1–2 during the entire operation of all three MBBR-MBR units [30]. On the optical microscope image in Figure 13b, it is shown that quite large aggregates and filamentous microorganisms exit into the filtrate. 

The size of aggregates in activated sludge at the first aerated tank of the bioreactors was reduced from 325 μm in control MBBR-MBR to 139 μm in ΜΒΒR-MBR K1. This is attributed to the strong movement of K1 biocarriers due to aeration, which prevents the forming of large sludge flocculates and changes the sludge morphology. In MBBR-MBR 13X-H, the size of the aggregates was reduced much less, and it reached 306 μm because the biocarriers agitation is much milder due to their increased weight.

In control MBBR-MBR, colloidal particles with ≤400 nm diameter, a diameter equal or less than the size of the membrane filtration pores, were observed to occupy 100% of the particles in the mixed liquor and the effluent until the 16th day of unit operation (Figure 14a). However, as the membrane fouling increased, the very small particles were reduced to a percentage less than 60–80%. In MBBR-MBR K1, fluctuations of the colloidal particle size were observed (Figure 14b), which can be explained by considering the respective fluctuations in the growing biofilm on the surfaces of the biocarriers (Table 2). The opposite trend was observed in MBBR-MBR 13Χ-H as compared to control MBBR-MBR. The colloidal particle concentration started low at 24% during the 5 days of unit operation and gradually increased reaching 80% on the 28th day of unit operation (Figure 14c). The fragmentation of biocarriers and the leakage of tiny particles of halloysite inside the unit can explain the above result and also the quick membrane fouling that took place. 

Figure 15 shows that biofilm was clearly produced not only inside the K1 biocarriers but also on the trabecular surfaces of the biocarriers’ walls. Based on the values for the dry mass of the biofilm shown in Table 2, it can be concluded that it gradually increased from 3.2 mg on the 6th day to 4.6 mg on the 32nd day. The dry mass of the biofilm was, however, reduced on the 41st day reaching 2.9 mg. A similar tendency is observed in the MLSS values. More specifically, their values changed increasingly from 40 mg/L on the 6th day of unit operation to 1100 mg/L on the 35th day. The MLSS value reduced to 390 mg/L on the 41st day. In both cases, this fluctuation is attributed to the easy biofilm detachment from the large holes of K1 biocarriers. Due to their large openings, biocarriers cannot hold the biofilm protected. The amount of SMP proteins that was found in the biofilm of K1 biocarriers was gradually increasing from 6 to 32 d (Figure 16). This increase matches the MLSS and biofilm increase on the surfaces of the biocarriers. Their decrease on the 35th to 40th days is linked to the biofilm detachment due to aeration. The same was also observed for the MLSS values. The SMP proteins and carbohydrates concentration was steadily low, less than 10 mg/L and 3 mg/L, respectively. The EPS proteins and carbohydrates concentration fluctuated in high concentrations with an average value equal to 90 mg/g TSS of EPS proteins and 40 mg/g TSS of EPS carbohydrates. No correlation between the growing biofilm on the biocarriers was observed. 

Biofilm was steadily grown on 3D-printed 13X biocarriers with halloysite, as shown in Figure 17. The large volume of biofilm is more easily observed in Table 3 than by optical observation, as the biofilm tends to develop on the inside surface of the biocarriers, which cannot be seen from the outside. According to Table 3, the dry mass of the biofilm values starts from 4980 mg on the 11th day of unit operation and steadily increases to 5711 mg on the 28th day of unit operation. Compared to the K1 Kaldnes biocarriers, the increase in the developed biofilm was three orders of magnitude larger, something that is due to the 3D-printed biocarrier design, which included very small holes and large inside depth. It is also observed that the biofilm is safely maintained inside the biocarriers and does not detach as a result of the strong aeration in the aerated tank. This was not observed in K1 commercial biocarriers. The respective results also come from the MLSS units’ values, which were 863 mg/L on the 11th day of unit operation and increased to 1250 mg/L on the 28th day of unit operation. The fluctuations are due to the aeration of the units, but they are negligible. The great increase in the developed biofilm on the 13X-H biocarriers is largely attributed to their construction material, halloysite, which is a highly promising filler, due to its high specific surface area, good dispersion, and excellent chemical and mechanical properties [38]. According to research by Zhang et al. (2021) [39], halloysite nanotubes displayed dramatically enhanced adsorption efficiency that could even remove tetracycline from wastewater, attributed to their high electrostatic attraction. 

The SMP proteins on the biofilm of the 13X-H biocarriers (Figure 18) increased by one order of magnitude and reached an average value of 29 mg/L as compared to the K1 biocarriers in which the average value was 7 mg/L (Figure 16). The same increase was also noted for the SMP carbohydrates (1.5 mg/L on the K1 biofilm and 17 mg/L on the 13X-H biofilm). On the contrary, the EPS proteins significantly decreased from an average value of 90 mg/g TSS on the biofilm of K1 biocarriers to an average value of 12 mg/g TSS on the 13X-H biocarriers. Similarly, the EPS carbohydrates reduced from an average value of 40 mg/g TSS on the K1 biocarriers to an average value of 13 mg/g TSS on the 13X-H. It is therefore concluded that EPS were mostly developed on the biofilm of the K1 biocarriers while more SMP were developed on the biofilm of 13X-H biocarriers than EPS. The increase in EPS might be attributed to the polyethylene material used for the fabrication of Κ1 biocarriers. According to the research of Yi et al. (2022) [40], the existence of polyethylene terephthalate (PET) microplastics in the wastewater treatment process increased EPS secretion, as a result of the stress to which biocarriers were subjected.

Furthermore, according to the results of TMP, the increase at the MBBR-MBR 13X-H (Figure 5) and the corresponding increase of colloidal particles in the mixed liquor (Figure 14), it may be concluded that it is due to the breakage of a small part of the 13X-H biocarriers, which corresponds to about 1/6 of their total volume. Fragmentation probably was caused due to the strong aeration on the inside of the first aerated tank (AT1). The biocarriers fragments are shown in Figure 19.

Regarding the microbiome analysis, the 16s rRNA sequencing analysis of the microbial communities growing on the tested biocarriers (Figure 20 and Figure 21) revealed that *Alphaproteobacteria* were the most dominant for both kinds of carriers. Indeed, this class has been previously shown to effectively grow and prevail on various biocarrier types [41]. Members of this group, as well as from *Betaproteobacteria*, which had comparable abundance in the K1 ring but quite lower in 3D printed carriers, are linked to enhanced COD reduction and participate in denitrification and phosphate accumulation [42]. *Actinobacteria* were also highly present in both types of carriers, mediating propionate acid fermentation, as well as acetate and H_2_ generation [43]. The most enriched genus found in the K1 ring was *Deinococcus* (*Deinococcaceae*), almost absent in 3D biocarriers 13X-H or sludge, which comprises a heterotrophic non-pathogenic aerobic group, able to survive extreme environmental conditions [44]. Though their role in the aerobic digestion of activated sludge has not been yet documented, various species can degrade different carbohydrates and demand minimal media for their growth [44]. *Mycobacterium* (*Mycobacteriaceae*), on the other hand, prevailed in the case of 3D carriers as opposed to K1 and was also present in the sludge. Many *Mycobacterium* species have developed an adaptive mechanism to increase their ability to degrade contaminants in challenging environments. This is achieved by the presence of mycolic acids in their cell walls, which facilitate the effective adhesion and interaction with contaminants, especially when they are highly hydrophobic, thus enhancing their biodegradation process [45]. Therefore, the presence of these organisms in the biofilm of 3D carriers may indicate improved organic contaminant removal from the system, contributing to overall process efficacy. Moreover, *Trichococcus*, which were copious in the sludge and 3D biocarriers, but barely detected in K1 ring, possess the ability to decompose benzene, an aromatic compound and common constituent of pesticides [46]. Consequently, it is possible that this bacterial group further promoted the biodegradation of pollutants in the system with 3D-biocarriers. It is worth noting, though, that *Nitrospira*, a major group of nitrite-oxidizing bacteria (NOB) [47], were more abundant in K1 ring than in 13X-H biocarriers. These bacteria have been previously reported to be the dominant NOB population in biofilms formed on K1 carriers used in combined systems of upflow blanket filter (UBF)-MBBR reactors [48].

## 5. Conclusions

In this study, a comparative evaluation of three MBBR-MBR units’ performance during municipal wastewater treatment was carried out, under the following conditions: when not adding biocarriers at all (control MBBR-MBR), when adding K1 biocarriers (MBBR-MBR K1), and when adding 13X-H biocarriers (MBBR-MBR 13X-H). In the control MBBR-MBR unit, as well as in the MBBR-MBR 13X-H, total membrane fouling was reached almost at the same period, on the 31st and 33rd day of operation, respectively. On the other hand, on the MBBR-MBR K1 unit, there was a notable improvement in the filtration, where the membrane fouling was reached 10 days later, on the 43rd day. The improvement of MBBR-MBR operation after biocarriers addition was expected since they incorporate the advantages of both suspended and attached biofilm growth. However, 13X-H biocarriers despite the many advantages of halloysite did not improve the membrane filtration, because of the fragmentation of a small part (1/6) of them. Regarding the efficiency in wastewater treatment, based on the COD values, it can be concluded that there is an excellent effluent quality in all three ΜΒΒR-MBR units, with a COD removal rate of 98%. Moreover, a clear improvement was achieved in nitrification and denitrification processes, after the addition of K1 and 13X-H biocarriers, where 57% and 55% of total N was removed, respectively, while in the case of the control MBBR-MBR, only 29% of total N was removed. Improving nitrification and denitrification processes is attributed to the biofilm formed on biocarriers that includes the existence of anoxic/anaerobic inner layers and aerobic outer layers. Finally, on 13X-H biocarriers, very high biofilm development was obtained, where dry mass increased by three orders of magnitude and ranged at 4980–5711 mg, compared to K1 biocarriers, where dry mass ranged at 2.9–4.6 mg. This result is largely attributed to the high specific surface area of halloysite as well as to the innovative design, where biofilm was safely maintained inside the biocarriers. 

## Figures and Tables

**Table 1:** Proportions of zeolite paste and clay binding medium.

	Material	Paste Content	Zeolite/Clay Percentage
Zeolite	13X	50%	89%
Inorganic binder	Halloysite nanotubes	6%	11%
Colloidal silica	Ludox AS-40	16%	
	Water	27%	
Organic binder	Methyl cellulose	1%	

**Table 2:** Dry mass and MLSS concentration values of the biofilm developed in the biocarriers in relation to the operating time of the unit.

t, d	Dry Mass of Biofilm, mg	MLSS, mg/L
6	3.2	40
20	3.5	240
32	4.6	360
41	2.9	20

**Table 3:** Dry mass and MLSS concentration values of the biofilm developed in the biocarriers in relation to the operating time of the unit.

t, d	Dry Mass, mg	MLSS, mg/L
11	4980	863
14	5426	1875
24	5210	1038
28	5711	1250

## Data Availability

Data presented in this study are available upon request from the corresponding author.
